# Frizzled-5 Receptor Is Involved in Neuronal Polarity and Morphogenesis of Hippocampal Neurons

**DOI:** 10.1371/journal.pone.0078892

**Published:** 2013-10-18

**Authors:** Paula G. Slater, Valerie T. Ramirez, Christian Gonzalez-Billault, Lorena Varela-Nallar, Nibaldo C. Inestrosa

**Affiliations:** 1 Centro de Envejecimiento y Regeneración (CARE), Departamento de Biología Celular y Molecular, Facultad de Ciencias Biológicas, P. Universidad Católica de Chile, Santiago, Chile; 2 Departmento de Biología, Facultad de Ciencias, Universidad de Chile, Santiago, Chile; National Cancer Center, Japan

## Abstract

The Wnt signaling pathway plays important roles during different stages of neuronal development, including neuronal polarization and dendritic and axonal outgrowth. However, little is known about the identity of the Frizzled receptors mediating these processes. In the present study, we investigated the role of Frizzled-5 (Fzd5) on neuronal development in cultured Sprague-Dawley rat hippocampal neurons. We found that Fzd5 is expressed early in cultured neurons on actin-rich structures localized at minor neurites and axonal growth cones. At 4 DIV, Fzd5 polarizes towards the axon, where its expression is detected mainly at the peripheral zone of axonal growth cones, with no obvious staining at dendrites; suggesting a role of Fzd5 in neuronal polarization. Overexpression of Fzd5 during the acquisition of neuronal polarity induces mislocalization of the receptor and a loss of polarized axonal markers. Fzd5 knock-down leads to loss of axonal proteins, suggesting an impaired neuronal polarity. In contrast, overexpression of Fzd5 in neurons that are already polarized did not alter polarity, but decreased the total length of axons and increased total dendrite length and arborization. Fzd5 activated JNK in HEK293 cells and the effects triggered by Fzd5 overexpression in neurons were partially prevented by inhibition of JNK, suggesting that a non-canonical Wnt signaling mechanism might be involved. Our results suggest that, Fzd5 has a role in the establishment of neuronal polarity, and in the morphogenesis of neuronal processes, in part through the activation of the non-canonical Wnt mechanism involving JNK.

## Introduction

The establishment and maintenance of neuronal polarity and the development of dendrites and the axon are pivotal steps in neuronal circuit formation and therefore crucial for central nervous system development. Acquisition of neuronal polarity in cultured neurons recapitulates cellular and molecular aspects of neuronal differentiation. Primary murine hippocampal neuron cultures are a very good model system for studying neuronal development, since it is a stereotyped process commanded by an intrinsic program [1,2]. At the extracellular milieu, several molecules can act as guidance cues for dendrite and axonal differentiation and elongation [3]. These guidance cues can be either attractive or repulsive, and include morphogens like Wnt glycoproteins [3,4]. 

In mammals, the Wnt family is composed of 19 Wnt proteins and 10 Frizzled (Fzd) receptors. The Fzd receptors are seven transmembrane-spanning receptors with a conserved cysteine-rich domain in the extracellular N-terminal region, where the Wnt ligands bind [5,6]. The binding of Wnt ligands to Fzd may activate different signaling cascades: the canonical Wnt/β-catenin signaling pathway and the non-canonical Wnt/Ca^+2^ and Wnt/c-Jun N-terminal kinase (JNK) pathways. When the Wnt/β-catenin pathway is activated, β-catenin accumulates in the cytoplasm and is translocated to the nucleus where it regulates the expression of target genes.The Wnt/Ca^+2^ pathway modulates the intracellular [Ca^+2^] and activates calcium sensitive proteins.The Wnt/JNK pathway activates monomeric G-proteins and kinases such as JNK, which affects cytoskeleton organization and dynamics [7,8,9]. A single Fzd receptor can be activated by more than one Wnt ligand, leading to different signaling cascades relying on the ligand-receptor pair and the cellular context (Mikels and Nusse, 2006). Therefore the study of Fzd receptors has become of great complexity and interest.

The Wnt signaling pathway has been shown to be involved in embryonic development and neuronal differentiation processes such as: polarity, migration, survival and morphogenesis, including length and branching of neurites [8,10,11]. Wnt-7a in cerebellar mossy fibers [12] and Wnt-3a in motoneurons [13], participate in the remodeling of axonal morphology. Wnt-5a stimulates axonal branching in sympathetic neurons [14], axonal growth in cortical cultures [15] and regulates dopaminergic neurite growth in a temporal dependent manner in ventral midbrain primary culture neurons [16]. While Wnt-7b generates an increase in the dendritic arbor complexity in hippocampal neurons [17]. Wnt signaling is also involved in synaptic development: e. i. Wnt-3a, Wnt-7a and Wnt-7b increase recycling and exocytosis of pre-synaptic vesicles [18,19]. Additionally, Wnt-5a modulates postsynaptic structure and function [20,21,22]. 

It has been reported that the expression and localization of Fzd receptors are highly regulated during hippocampal development [23]. Moreover, some Fzd receptors have been related to axonal growth in the case of Fzd3 [24], and to synapse formation in hippocampal neurons in the case of Fzd1 [25] and Fzd5 [26]. However, the identity of Fzd receptors that mediate early events regulated by the Wnt pathway still remains elusive. Interestingly, Fzd5 has been described as one of the receptors for Wnt-5a and Wnt-7a and these both ligands have roles on early neuronal development such as axonal development and remodeling [26,27,28]. Also, Fzd5 is expressed in the brain since early postnatal development [26] and participates on neural potentiality in the developing retina [27,29], making this receptor a suitable focus of study. In the present work, we investigated the expression, distribution and potential role of Fzd5 in early stages of hippocampal neurons culture. Our findings suggest that Fzd5 is involved in the establishment of neuronal polarity and in neuronal morphogenesis in part through a non-canonical Wnt-JNK-dependent pathway. 

## Materials and Methods

### Ethics Statement

Sprague-Dawley rats used in these experiments were housed at the Faculty of Biological Sciences of the P. Universidad Católica de Chile and handled according to guidelines outlined and approved by the Institutional Animal Care and Use Committee (IACUC) at the Faculty of Biological Sciences of the P. Universidad Católica de Chile; and in accordance with the main directions from the Science and Technology National Commission (CONICYT) about experimental animal research. Animals were euthanized by anesthesia overdose. In our case, the IACUC did not require approval of specific protocols, because housing and handling procedures, as well as euthanasia, are well standardized protocols conceived and constantly monitored by IACUC itself. No experimental procedures were performed on the rats prior to euthanasia.

### Primary culture of rat hippocampal neurons

Rat hippocampal cultures were prepared from Sprague-Dawley rats, of either sex, at embryonic day 18, as previously described [30,31]. On day two, cultured neurons were treated with 2 μM cytosine arabinoside for 24 h; this method resulted in cultures highly enriched of neurons (approximately 5% glia). 

### Neuronal, HEK293 and PC12 cells transfection

Neurons were co-transfected using Lipofectamine^TM^ 2000 (Invitrogen, Carlsbad, CA, USA). For the overexpression experiments, neurons were transfected with 0.4 μg of Fzd5-HA or PCS2^+^ plasmid plus 0.1 μg of GFP vector, one, two or three days after seeding onto poly-L-lysine coated cover slips in 24-well culture plates at a density of 4 × 10^4^ cells per well. Loss-of-function experiments were achieved using SureSilencing^TM^ shRNA plasmids. Neurons were transfected with 0.5 μg of a equimolar mixture of four shRNA Fzd5 plasmids (SABiosciences, Frederick, MD, USA), one day after plating onto poly-L-lysine-coated cover slips in 24-well culture plates at a density of 4 × 10^4^ cells per well. 

HEK293 cells (American Type Culture Collection (ATCC), Manassas, VA, USA) were co-transfected with 1.72 μg of Fzd5-HA or PCS2^+^ plasmid plus 0.58 μg of GFP using Lipofectamine^TM^ 2000 (Invitrogen) 24 h after seeding in six-well culture plates at a density of 1.6 × 10^6^ cells per well.

PC12 cells (American Type Culture Collection (ATCC)) were co-transfected with 2 μg of an equimolar mixture of the four shRNA Fzd5 plasmids using Lipofectamine^TM^ 2000 (Invitrogen) 24 h after plating in six-well culture plates at a density of 1.6 × 10^6^ cells per well.

### Immunofluorescence

Hippocampal neurons were rinsed twice in ice-cold PBS and fixed with a freshly prepared solution of 4 % paraformaldehyde plus 4% sucrose in PBS for 20 min and permeabilized for 5 min with 0.2 % TritonX-100 in PBS. After several rinses in ice-cold PBS, cells were incubated in 1 % bovine serum albumin (BSA) in PBS (blocking solution) for 30 min at 37 °C, followed by an overnight incubation at 4 °C with primary antibodies. Cells were thoroughly washed with PBS and then incubated with Alexa-conjugated secondary antibodies (Molecular Probes, Carlsbad, CA, USA) for 1 h at 37 °C. Coverslips were mounted in Fluoromont G mounting media and analyzed on an Olympus Takyo Japan Fluoview FV 1000 confocal microscope. Primary antibodies used were goat anti-Fzd5 (Santa Cruz Biotechnology Inc., Santa Cruz, CA, USA), mouse anti-MAP1B-P, clone SMI31 (Covance, Princeton, NJ, USA), rabbit anti-MAP2 (Millipore, Billerica, MA, USA), mouse anti-Tau1 (Millipore), mouse anti-β-III-tubulin (Promega, Madison, WI, USA) and phalloidin labeled with ^633^Alexa (Molecular Probes). Images were analyzed using NIH ImageJ software. Dendritic and axonal branching quantification were carried out manually. Length measurements were carried out under threshold conditions using the NIH ImageJ software. 

### Sholl analysis

Sholl analysis was performed using the NIH ImageJ software with the sholl analysis plug-in that consists of a semi-automated program in R, in which the soma boundary is approximated by an ellipsoid and dendrite intersections were assessed at radial distances of 5 μm from the soma [32,33]. 

### Immunoblot analysis

Extraction of total protein from brain and hippocampi of Sprague-Dawley rats, cell culture of hippocampal neurons and of HEK293 and PC12 cell line, and immunoblot analysis were carried out as previously described [25]. Primary antibodies used were goat anti-Fzd5 (Santa Cruz Biotechnology Inc., R&D Systems, Minneapolis, MN, USA) and rabbit anti-Fzd5 (Abcam, Cambridge, MA, UK), mouse anti-PSD95 (UC Davis/NIH NeuroMab Facility, Davis, CA, USA), mouse anti-β-actin (Sigma-Aldrich, St Louis, MO, USA), rabbit anti-N-cadherin (Santa Cruz Biotechnology Inc.), rabbit anti-pJNK (Cell Signaling Inc., Beverly, MA, USA), rabbit anti-JNK (Cell Signaling Inc), rabbit anti-β-tubulin (Santa Cruz Biotechnology Inc), rabbit anti-pGSK-3βSer9 (Cell Signaling Inc), mouse anti-pGSK-3βTyr216 (Becton Dickinson, San Jose, CA, USA), mouse anti-GSK-3β (Santa Cruz Biotechnology Inc), rabbit anti-c-jun (Santa Cruz Biotechnology Inc), rabbit anti-p-c-Jun (Cell Signaling Inc), mouse anti-GFP (NeuroMab Facility), rabbit anti CRMP2 (Abcam) and rabbit anti-HA (Santa Cruz Biotechnology Inc). 

### Statistical analysis

Statistical analysis was performed using the statistical software Prism 5 (GraphPad Software Inc., San Diego, CA, USA). Values are expressed as mean ± standard error of the mean. Statistical significance of differences was assessed with the paired Student's *t*-test or ANOVA with Bonferroni post-test (*P* < 0.05 was considered significant).

## Results

### Fzd5 receptor is expressed early and shows a polarized distribution in developing hippocampal neurons

**Figure 1:**
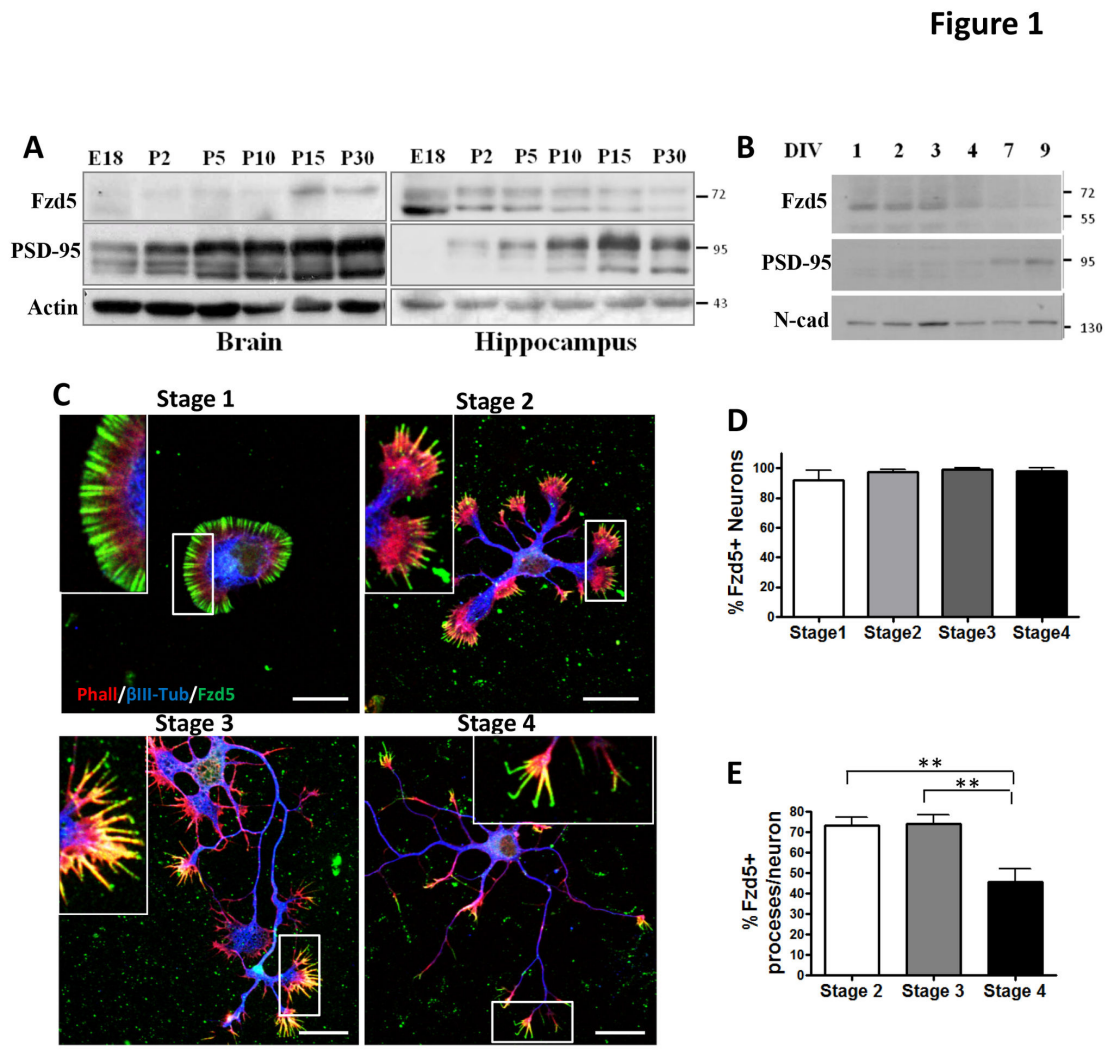
Expression and distribution of Fzd5 during development of cultured hippocampal neurons. **A**. Detection of Fzd5 protein levels in total brain and hippocampi homogenates. The receptor levels in brain homogenates augmented concomitantly with the progression of postnatal days, on the contrary, in hippocampi homogenates the expression of Fzd5 diminished through development. **B**. Detection of Fzd5 protein levels in total homogenates of cultured hippocampal neurons after different days in vitro (DIV). The receptor was detected from 1 DIV and its levels were maintained until 4 DIV. **C**. Neurons were classified in different developmental stages [1] and the distribution of Fzd5 was analyzed. At stage 1 the receptor was localized at the distal part of the lamellipodia surrounding the neuron and in the rest of the stages at the tips of the filopodia present in the growth cone. Magnifications of the region (inset) are seen in the upper corner. **D**. Quantification of the percentage of neurons positive for Fzd5 at each stage of development. More than 90 % of neurons were positive for Fzd5 in the different developmental stages. **E**. Quantification of the percentage of processes per neuron positive for Fzd5 at each stage. Scale bar: 20 µm. Bars represent mean ±SEM of three independent experiments. ** p<0.001.

The development of hippocampal neurons in culture has been divided into five discrete stages (Dotti et al., 1988). Neurons form actin-dependent lamellipodia shortly after plating onto an adherent surface (stage 1) and develop several minor neurites that extend from the cell body displaying equivalent lengths (stage 2). Neuronal symmetry is broken when one of the minor neurites extends more prominently than its siblings to become the axon (stage 3), and then the rest of the minor neurites develop into dendrites (stage 4). Finally, dendritic maturation occurs with the appearance of dendritic spines, specialized structures involved in neurotransmission (stage 5). The subcellular distribution and the role of Fzd5 on hippocampal neurons have been examined on stage 5 neurons, during synaptogenesis [26], however Fzd5 functions in early stages of neuronal development are unavailable. In order to determine whether Fzd5 participates at initial stages of neuronal development, the expression of Fzd5 was examined in brain and hippocampus homogenates from different embryonic and postnatal stages. In whole brain homogenates the levels of Fzd5 were very low at embryonic day 18 and increased during the postnatal period (Figure 1A) as previously reported [26]. Fzd5 expression increased in parallel with the appearance of the post-synaptic density protein (PSD-95) [34]. Interestingly, in hippocampal homogenates, a different profile for Fzd5 expression was observed. Fzd5 was robustly detected at embryonic day 18 and in early postnatal stages and its expression decreased after postnatal day 15 (Figure 1A). Analyses of Fzd5 expression were carried out since embryonic day 18 (E18) because the hippocampal neuron cultures used in the present study are prepared from rats at that embryonic stage as previously described [35]. However, since the higher expression of Fzd5 was observed on E18, the expression profile of Fzd5 was examined more extensively during earlier hippocampal development. Thus, Fzd5 expression was analyzed in hippocampus homogenates obtained from E17 - E19 rats. The peak of Fzd5 expression was observed on E17, and started to diminish thereafter (Figure S1).

The expression and distribution of Fzd5 was then studied in cultured hippocampal neurons recapitulating the developmental stages, as days in vitro (DIV), described for the acquisition of neuronal polarity and subsequent neuronal maturation (Dotti et al., 1988). Immunodetection of Fzd5 in protein homogenates from hippocampal neurons between 1-9 DIV revealed that Fzd5 was expressed as early as 1 DIV and its expression was maintained until DIV 4, and its expression decreases to very low levels thereafter (Figure 1B). As expected, PSD-95 began to be expressed at 7 DIV and increased at 9 DIV (Figure 1B). N-cadherin was evenly expressed throughout the duration of the time-course (Figure 1B). 

The sub-cellular localization of Fzd5 was analyzed during neuronal development by immunofluorescence using an anti-Fzd5 antibody directed against the C-terminal region of the protein. Fzd5 showed diffused distribution in the soma and in actin-rich structures localized distally in the neuron. The specificity of Fzd5 staining was confirmed using another antibody directed against the N-terminal region of the receptor (Figure S2). By quantitative analyses it was determined that virtually all neurons were positive for Fzd5 staining from stages 1 to 4 (Figure 1D). At stage 1, Fzd5 was located in lamellipodia structures surrounding the neurons (Figure 1C, top left). As development progresses and neurites began to extend (stages 2 to 4), Fzd5 staining was restricted to neurite growth cones, where it decorated actin-based filopodia structures stained with phalloidin (Figure 1C, magnifications). Fzd5 was present in 73.1 ± 6.4 % of neurites per neuron at stage 2 and 74 ± 6.7% at stage 3 (Figure 1E). At stage 4, the percentage of neurites per neuron positive for Fzd5 decreased significantly, to 46 ± 8% (Figure 1C, bottom right and Figure 1E). 

**Figure 2:**
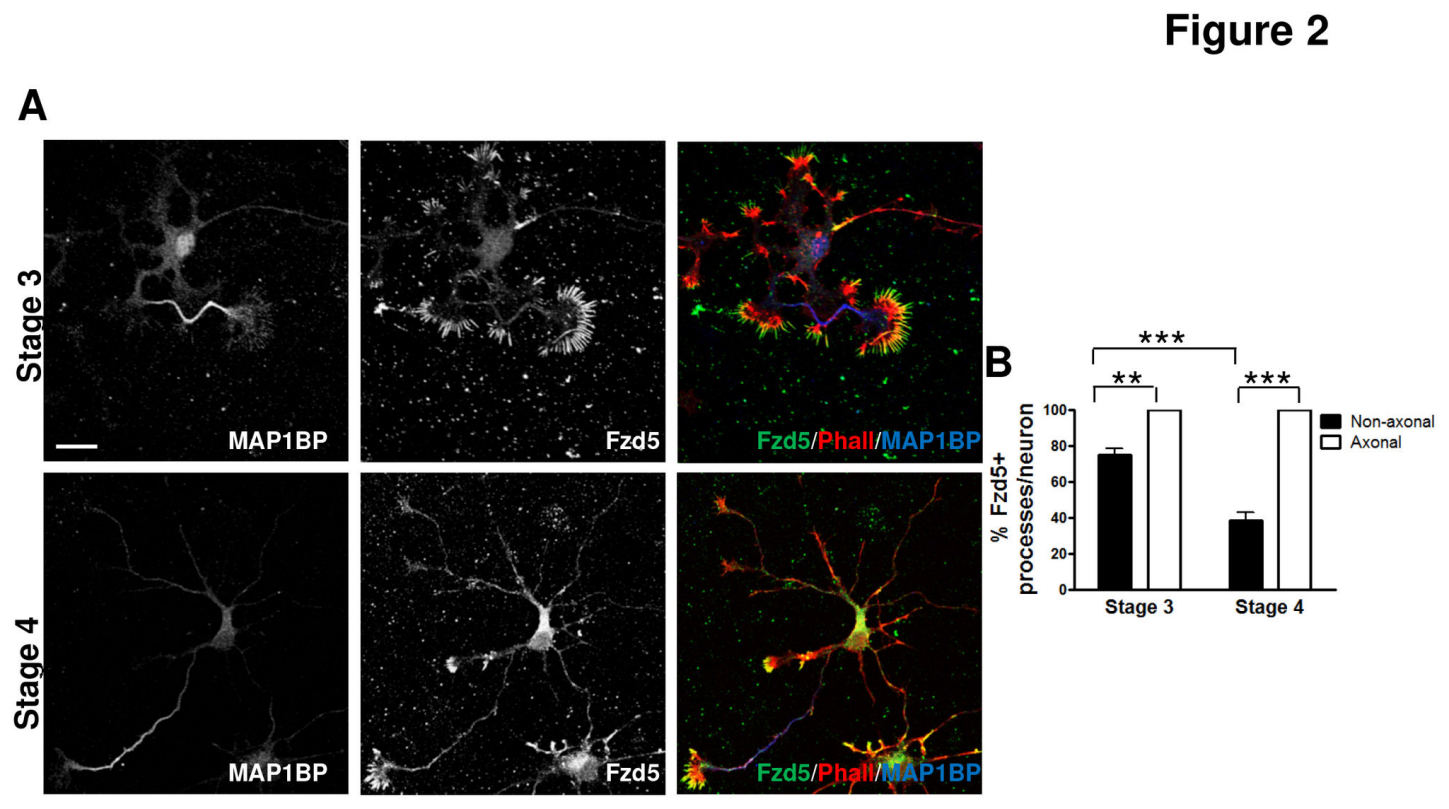
Fzd5 polarizes towards the axon. **A**. Immunodetection of Fzd5 in neurons at developmental stages 3 and 4. MAP1B-P was used to identify the axonal processes and Phalloidin staining to observe F-actin distribution and identify growth cones. Scale bar: 20 µm. **B**. Quantification of Fzd5-positive axonal and non-axonal processes in neurons at stages 3 and 4. All axons were positive for Fzd5 (Fzd5+) in both stages, but the percentage of non-axonal Fzd5+ processes declined throughout development. Scale bar: 10µm. Bars represent mean ±SEM of three independent experiments. ** p< 0.01; *** p< 0.001.

As the percentage of neurons positive for Fzd5 staining was nearly 100% in stage 3 and 4, and the percentage of neurites positive for Fzd5 staining per neuron diminished in stage 4 neurons, the identity of the neurites loosing Fzd5 staining was evaluated. The co-distribution of Fzd5 with the axonal marker, phosphorylated MAP1B (MAP1B-P), was evaluated in neurons at stages 3 and 4 (Figure 2A, B). All neurons had axons positive for Fzd5 staining at both developmental stages (Figure 2A, B, left panels). In contrast, a 75 ± 5.7 % of non-axonal processes per neuron determined as neurites negative for MAP1B-P, were positive for Fzd5 at stage 3 and this percentage decreased to 39 ± 5.6 % at stage 4 (Figure 2B). Indicating that Fzd5 expression was maintained in the axons but decreased in non-axonal processes as the neurons progressed from stage 3 to stage 4. These results suggest that Fdz5 tends to accumulate in axons during neuronal maturation.

### Fzd5 receptor participates in the establishment of neural polarity

It is known that several molecules that stimulate axon development are primarily polarized to the axon, initiating a positive feedback loop necessary for the axonal establishment [36,37]. In the present work it was shown that Fzd5 followed a similar pattern of expression. So, in order to determine whether Fzd5 could have a role in the establishment of neuronal polarity, the distribution of different axonal markers was evaluated in neurons displaying gain- and loss-of-function for Fzd5. 

**Figure 3:**
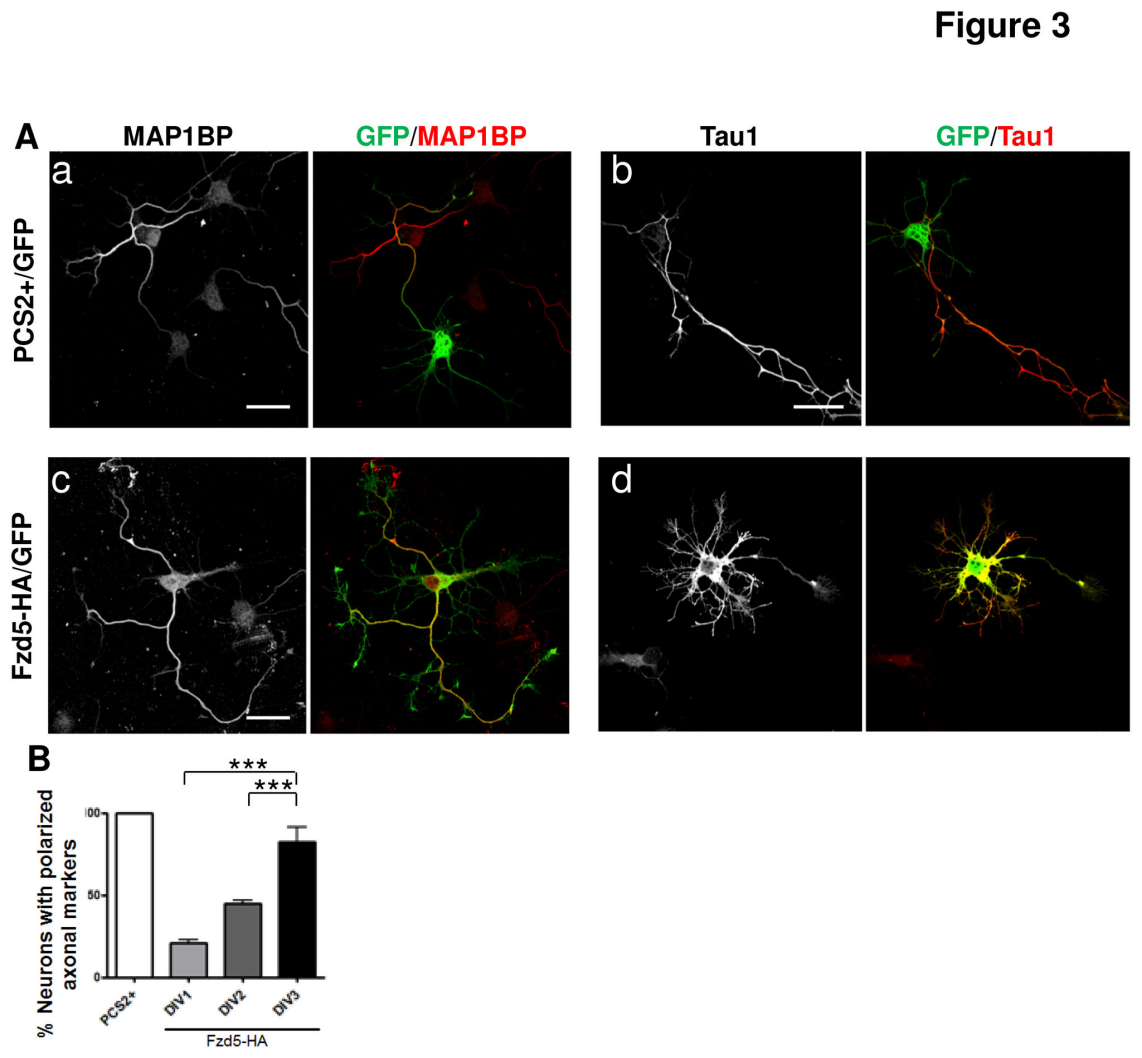
Fzd5 overexpression alters the polarity of hippocampal neurons. **A**. Immunodetection of the axonal markers MAP1B-P and Tau1 in neurons 24 h after transfection with the empty vector PCS2+/GFP as a control or Fzd5-HA/GFP at 2 DIV. GFP was used to identify transfected neurons. Representative images, shown in panels a and b, of control neurons expressing the axonal markers distributed in one neurite and in panels c and d, neurons that overexpress Fzd5 with the axonal marker distributed in the whole cell. **B**. Quantification of neurons transfected on 1-3 DIV with polarized distribution of an axonal marker. The percentage of neurons with polarized axonal markers was significantly lower in neurons overexpressing Fzd5 than in control neurons, in a DIV-dependent manner. Scale bar: 20 µm. Bars represent mean ±SEM of three independent experiments. *** p< 0.001.

For the gain-of-function approach, neurons at 2 DIV were co-transfected with Fzd5-HA plus GFP or with the empty vector PCS2+ plus GFP as control. Twenty-four hours post-transfection, neurons were fixed and immunostained with an antibody against Fzd5 and the neuronal marker β-III-tubulin. Transfected neurons were identified with GFP. As expected, a strong increase in Fzd5 staining was observed in neurons transfected with Fzd5 (Figure S3, yellow arrow), as compared to non-transfected neurons (Figure S3, white arrow), however the polarized distribution of Fzd5 was lost and the receptor was detected equally distributed in the whole cell (Figure S3). To evaluate the effect of Fzd5 overexpression on neuronal polarity, the distribution of the axonal markers Tau1 and MAP1B-P were analyzed 24 h post-transfection. Control GFP+ neurons showed positive staining for Tau1 and MAP1B-P in only one neurite, as expected for classic axonal marker distribution (Fig. 3Aa, Ab) but, interestingly more than 50 % of neurons overexpressing Fzd5 showed staining for Tau1 or MAP1B-P (Fig. 3Ac, Ad and 3B) in all neurites, suggesting that these neurons have impaired polarity. At 2 DIV it is expected that an important percentage of neurons have already developed an axon so, the effect of overexpression of Fzd5 was evaluated earlier in development. Neurons were transfected at 1 DIV and analyzed at 3 DIV. Almost 80 % of Fzd5-overexpressing neurons lost polarized distribution of the axonal markers (Figure 3B). Finally, it was evaluated whether Fzd5 overexpression could also affect axonal marker distribution in already polarized neurons. Therefore, neurons were transfected at 3 DIV, when almost all neurons are at stage 3 and have developed an axon. No differences were observed in the distribution of axonal markers between control and neurons overexpressing Fzd5 (Figure 3B). These results showed that Fzd5 overexpression affects the distribution of axonal markers during the early stages of neuronal development, but neurons that have already developed an axon are unaffected, suggesting that Fzd5 may be more important for the initial stages of neuronal polarity. 

**Figure 4:**
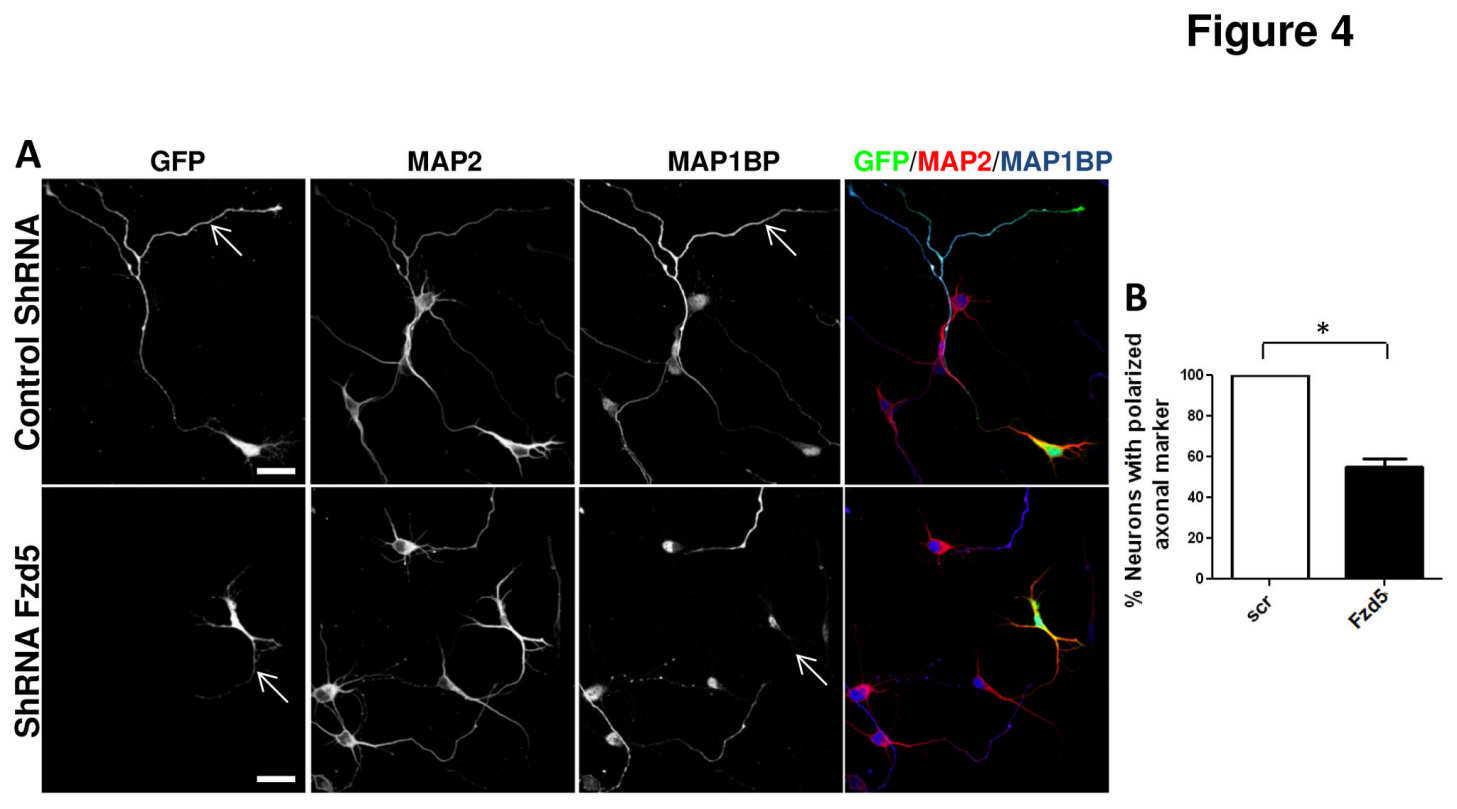
Loss-of-function of Fzd5 alters the polarity of hippocampal neurons. **A**. Immunodetection of the axonal marker MAP1B-P and the somato-dendritic marker MAP2 in neurons 48 h after transfection with the scramble or shFzd5 vectors. GFP was used to detect transfected neurons. Representative images, shown in the upper panels, of a control neuron expressing the axonal marker distributed through the axon (arrows) and in the lower panels, a neuron transfected with the shFzd5 expressing no axonal marker (arrows). Scale bar: 20 µm. **B**. Quantification of transfected neurons with polarized axonal marker. Polarization of axonal markers was significantly lower in neurons transfected with the shFzd5 vectors compared to that of control neurons. Bars represent mean ±SEM of three independent experiments. * p< 0.05.

As shown by gain-of-function experiments, the neurons transfected on DIV 1 were the most affected by overexpression of Fzd5. So, we decided to evaluate the effect of the loss-off-function of Fzd5 on 1 DIV neurons. To decrease Fzd5 expression, hippocampal neurons were incubated with an equimolar mixture of 4 Fzd5 shRNAs or scramble vectors [26], these plasmids also contained the GFP gene to identify transfected cells. Fzd5 knock-down by shRNAs was evaluated by immunofluorescence in cultured hippocampal neurons and, because of the low transfection efficiency in neurons, by western blot in the rat cell line PC12. As expected, both immunofluorescence (Figure S4A) and western blot (Figure S4B) analyses showed that Fzd5 expression was diminished in cells transfected with shRNA plasmids. Figure S3A shows a representative image of neurons transfected with shRNA and immunostained with an antibody against Fzd5 and the neuronal marker β-III-tubulin. Neurons transfected with the shRNA (GFP+ and β-III-tubulin+) showed non-staining for Fzd5 (Figure S4A, white arrow), while neurons in the same culture that were not transfected (GFP- and β-III-tubulin+) showed the usual staining for Fzd5 (Figure S3A, yellow arrow). Western blot of PC12 cell extracts showed a decrease of the band observed at 72 KDa corresponding to Fzd5, in cells transfected with shRNA plasmids compared to cells transfected with the scramble shRNA (Figure S4B). The levels of GFP expression were similar in both conditions, suggesting a similar transfection efficiency. In addition, Fzd5 knock-down by these shRNAs was previously determined in a different rat cell line and also in cultured hippocampal neurons (Sahores et al., 2010). Hippocampal neurons at 1 DIV were co-transfected with 4 Fzd5 shRNA or scrambled plasmids, and the distribution of the axonal marker MAP1BP and the somato-dendritic-specific microtubule associated protein MAP2 [38] were analyzed 24 h post-transfection (Figure 4A). In order to assess the neuronal identity of transfected cells, GFP+ cells, also positive for MAP2, were selected for analyses. Scramble GFP+ neurons showed that the axonal marker MAP1BP was positive in only one neurite (Figure 4A, upper panels), as expected, but only 53.33% of the shRNA expressing neurons showed this distribution, the other 46.67% showed no axonal marker expression (Figure 4A, lower panels and 4B), that is to say that almost 50% of the neurons were not polarized, supporting the suggested role of Fzd5 in neuronal polarity establishment. 

### The inhibition of JNK, partially prevented neuronal polarity alterations induced by Fzd5 gain of function

**Figure 5:**
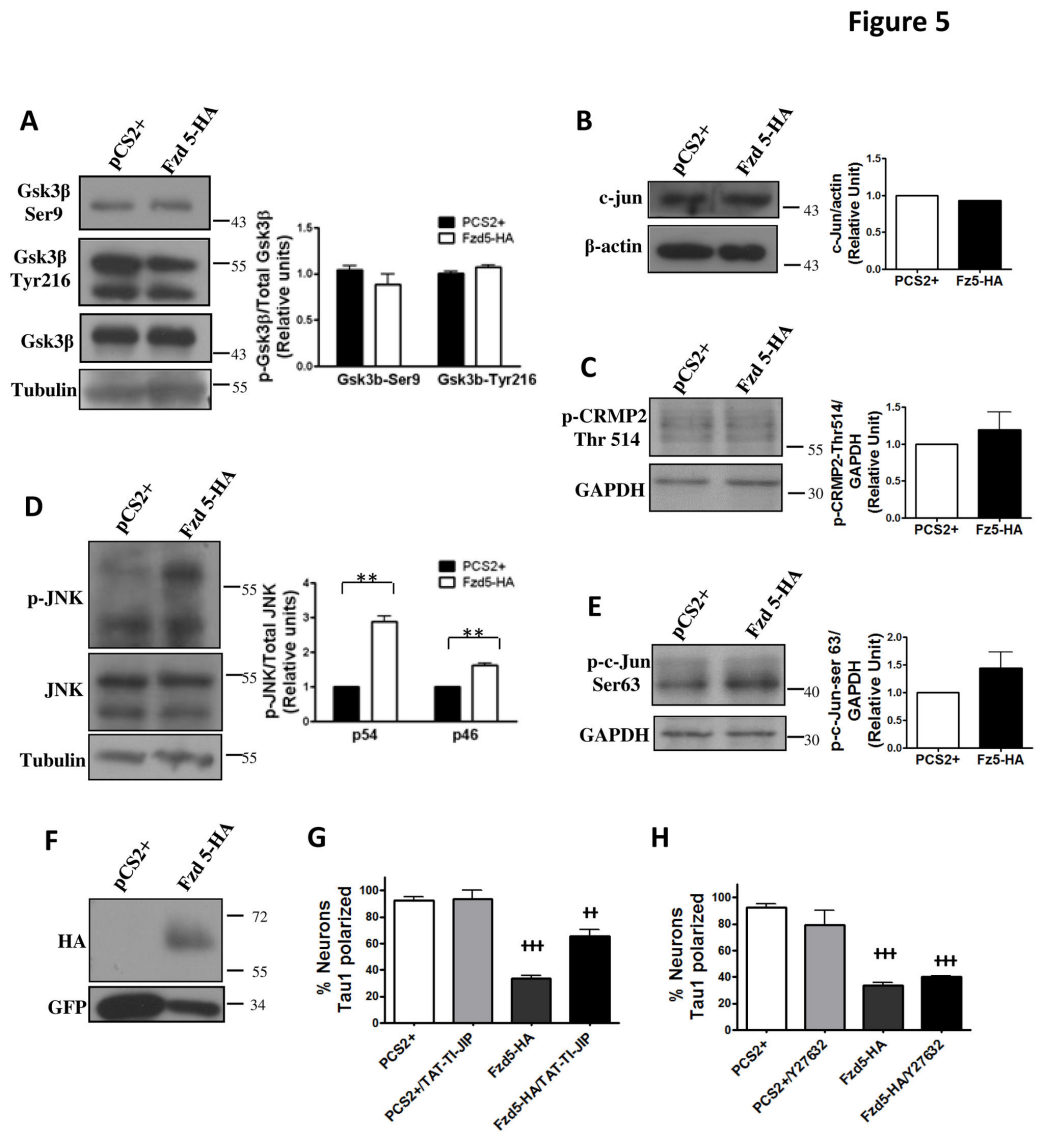
Overexpression of Fzd5 activates JNK in HEK293 cells and the effects of Fzd5 overexpression on neuronal polarity are partially prevented by the JNK inhibiton. **A**-**C**. Detection of main effectors of canonical Wnt pathway in homogenates form HEK293 cells transfected with Fzd5-HA or the empty vector pCS2+. Activated GSK-3β (pGSK-3βTyr216), inhibited GSK-3β (pGSK-3βSer9), total GSK-3β (A), c-jun (B) and p-CRMP (C) levels were very similar in control and overexpressing Fzd5 cells. **D**-**E**. Detection of activated JNK levels in homogenates form HEK293 cells transfected with Fzd5-HA or the empty vector pCS2+. Cells overexpressing Fzd5 presented increased levels of activated JNK (pJNK) isoforms (D) and p-c-Jun (E) compared to control cells. **F**. Detection of HA and GFP in HEK293 homogenates as control of transfections. **G**. Quantification of neurons with a Tau1 polarized distribution after treatment with JNK inhibitor. The percentage of cells with polarized axonal markers was significantly lower in neurons overexpressing Fzd5 compared to controls, and was in part prevented by treatment with TAT-TI-JIP. **H**. Quantification of neurons with polarized distribution of Tau1 after treatment with Rho inhibitor. The percentage of neurons with polarized axonal markers was significantly lower in cells overexpressing Fzd5 compared to controls;Y27362 treatment had no effect. Bars represent mean ±SEM of four independent experiments. In the graphs, JNK2/3: 55 kDa and JNK1: 46 kDa.

Actin and microtubule dynamics are essential for the initiation, extension and maintenance of axons and dendrites [37,39,40]. It has been described that both, canonical and non-canonical Wnt pathways participate in regulating cytoskeleton dynamics and organization [7,41] and interestingly Fzd5 has been implicated in both signaling cascades [27]. On one hand, the activation of the non-canonical Wnt/JNK pathway enhances Rho family GTPases and JNK activity [7,8]. Rho has been implicated in cytoskeleton changes involved in limiting dendrite branching [42] and also in axonal branching [43]. Rac1 is also necessary for axonal development [44,45,46], and activates JNK, which generates microtubule stability and is involved in dendrite formation [17,47]. GSK-3β participates in the canonical Wnt signaling pathway, regulating microtubule associated proteins activity [41]. In the next set of experiments we assessed the ability of Fzd5 to activate these Wnt signaling cascades. Activation of canonical Wnt and Wnt/JNK pathways were evaluated by western blot in HEK293 cells co-transfected with Fzd5-HA plus GFP or with the empty vector PCS2+ plus GFP as control. Two components of the canonical Wnt signaling pathway were evaluated: the activity of GSK-3β and the levels of the target gene c-jun [48]. The activation of GSK-3β was evaluated using a specific antibody against GSK-3β phosphorylated on tyrosine 216 (pGSK-3βTyr216), and the inhibition of the enzyme was evaluated with an antibody directed against GSK-3β phosphorylated on serine 9 (pGSK-3βSer9). Also, to assess the activity of GSK-3β, the phosphorylation levels of collapsing response mediator protein 2 (CRMP2) on threonine 514, one of its target protein, was analyzed. Non-canonical Wnt/JNK pathway was evaluated assessing the activation of JNK by using an antibody against phosphorylated-JNK (pJNK) and, analyzing the phosphorylation levels of the JNK target protein c-Jun on serine 63. HEK293 cell transfected with Fzd5-HA did not show significant differences on pGSK-3βSer9 or pGSK-3βTyr216 compared with control cells (Figure 5A). Moreover, no changes in the total levels of c-jun (Figure 5B) or p-CRMP2 (Figure 5C) were detected. In contrast, overexpression of Fzd5-HA significantly increased all the pJNK isoforms, 55kDa corresponding to JNK2/3 and 46kDa corresponding to JNK1, without changing the overall levels of JNK (Figure 5D). Also a tendency of increased levels of p-c-Jun was also observed (Figure 5E). These results suggest that Fzd5 might be capable of activating Wnt/JNK pathway.

To evaluate if the effect of Fzd5 overexpression on neuronal polarity is due to the activation of Wnt/JNK pathway, inhibitors of JNK and Rho kinase, two main components of Wnt/JNK pathway were used. Neurons at 2 DIV were co-transfected with Fzd5-HA plus GFP or with the empty vector PCS2+ plus GFP as a control, and were treated 2 h after transfection with or without 1 µM of the JNK inhibitor, TAT-TI-JIP (Calbiochem, Darmstadt, Germany), which is a small 11-mer peptide of JIP conjugated to Tat peptide, that affects the JNK3 activity [49], or with 37 µM of the Rho kinase inhibitor, Y27632 (Calbiochem) [50]. Twenty-four hours later, neurons were fixed and immunostained for the axonal marker Tau1 and somato-dendritic marker MAP2. A 32.7 % of Fzd5-overexpressing neurons showed a polarized distribution of Tau1, and this negative effect in polarization was partially prevented in neurons treated with TAT-TI-JIP, were 65.4 % of neurons showed a polarized distribution of the axonal marker (Figure 5E). The Rho inhibitor had no effect on polarity impairment produced by Fzd5 (Figure 5F). These results suggest that the impaired polarity induced by Fzd5 overexpression involves, in part, Wnt/JNK signaling activation.

### Fzd5 overexpression in stage 3 neurons alters neuronal morphogenesis

**Figure 6:**
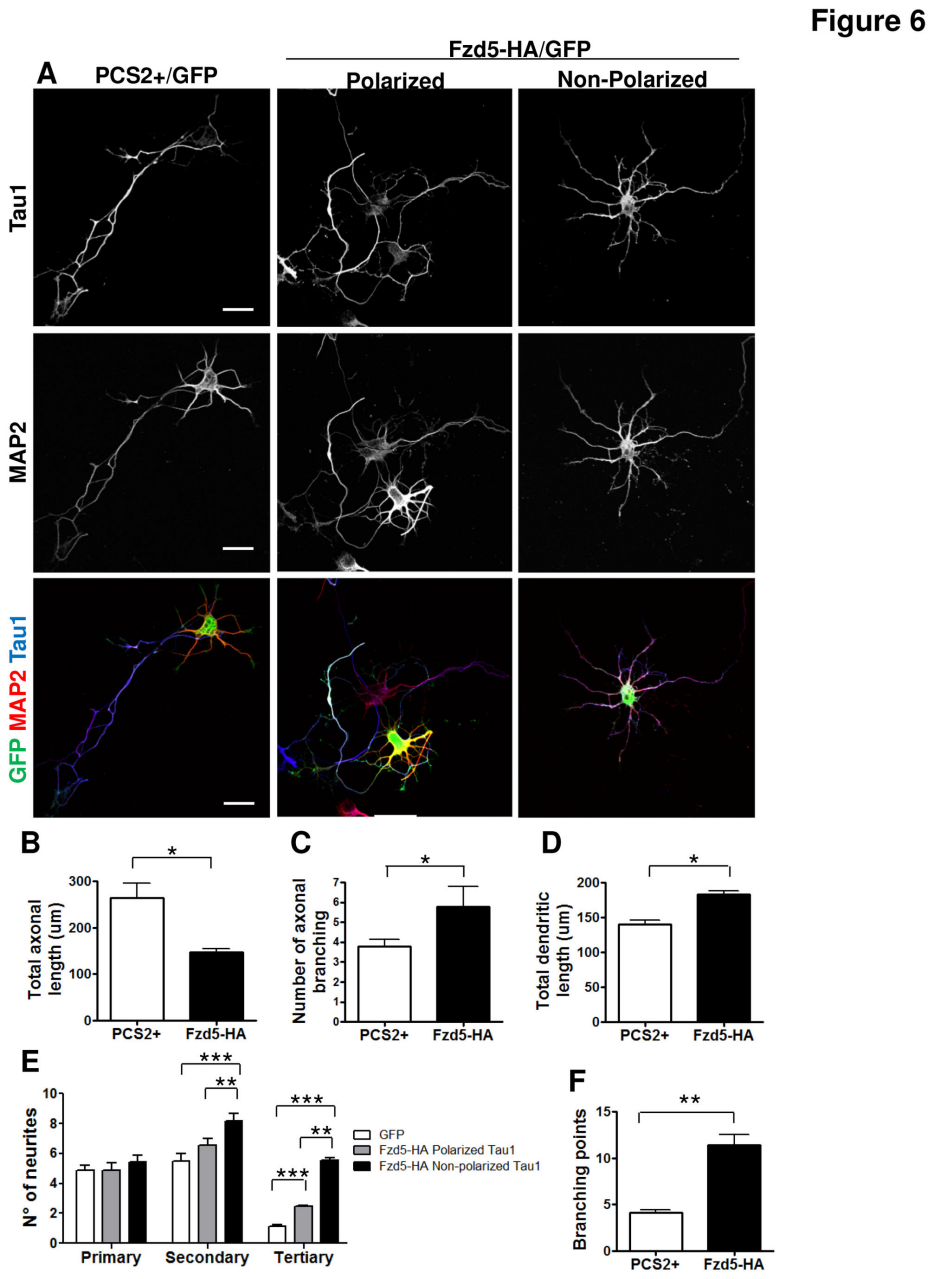
Fzd5 overexpression in neurons that have already developed the axon alters the morphology of hippocampal neurons. **A** Immunodetection of transfected (GFP+) hippocampal neurons with the axonal marker Tau1 (upper panels) and the somato-dendritic marker MAP2 (middle panels) in neurons transfected with the empty vector or overexpressing Fzd5 at 3 DIV. In the lower panels a merge of both markers with GFP is shown. **B**-**D**. Quantification of axonal length (D), total axonal branching (E), and total dendritic length (F). Neurons overexpressing Fzd5 with polarized axonal markers distribution showed a significant decrease in axonal length and a significant increase in axonal branching and dendritic length, compared to control neurons. **E**. Quantification of primary, secondary and tertiary dendritic branching. Neurons overexpressing Fzd5, with non-polarized axonal markers, showed a significant increase in secondary and tertiary dendritic branching compared to control neurons. Polarized neurons overexpressing Fzd5 showed significantly more tertiary branching than control neurons. **F**. Quantification of branching points of the neurites. Neurons overexpressing Fzd5 showed a significant increase in branching points compared to control neurons. Scale bar: 20 µm. Bars represent mean ±SEM of three independent experiments. * p< 0.05; ** p< 0.01; *** p< 0.001.

There is evidence of Wnt ligands participating in neuronal morphogenesis after the establishment of neuronal polarity [15,51,52], therefore we studied whether Fzd5 overexpression also affected axonal and dendrite morphology in neurons that had already developed an axon. For this analysis we used neurons transfected at 2 or 3 DIV and only considered neurons with neuronal polarity unaffected by Fzd5 overexpression, which corresponded to neurons that had developed the axon before transfection (Figure 6A, middle panels). The analyses were done 24 h post-transfection. In neurons overexpressing Fzd5, the axonal length significantly decreased from 263.62 ± 32.13 to 146.95 ± 7.65 µm (P<0.05) when transfected at 2 DIV (Figure 6B) and from 319.24 ± 10.39 to 142.02 ± 24.9 µm (P<0.01) when transfected at 3 DIV (data not shown). On the other hand, the number of axonal branches increased from 3.8 ± 0.34 to 5.78 ± 1.02 (P<0.05) in neurons transfected at 2 DIV (Figure 6C) and at 3 DIV from 2.68 ± 0.22 to 5.22 ± 0.31 (P<0.01) (data not shown). These findings suggest that Fzd5 participates in axon morphogenesis, after the establishment of the axon. 

Dendritic length was also analyzed by means of measuring the length of neurites positive for MAP2 , and a significant increase in dendritic length was observed from 140.24 ± 6.42 to 182.96 ± 5.46 µm (P<0.05) in neurons transfected at 2 DIV (Figure 6D) and from 187.02 ± 33.19 to 320.91 ± 32.19 µm (P<0.01) in neurons transfected at 3 DIV (data not shown). Finally, the role of Fzd5 in dendritic arborization was examined (Figures 6E, F, S4). The number of primary, secondary and tertiary neurites was quantified in neurons transfected at 2 DIV, neurons with a polarized and non-polarized phenotype were considered (Figure 6A, E). Polarized neurons (P) overexpressing Fzd5 showed a significant increase in the number of tertiary dendrites (control: 1.13 ± 0.13; Fzd5_P_: 2.45 ± 0.07, P<0.001) and non-polarized (NP) neurons overexpressing Fzd5 showed a significant increase in secondary and tertiary dendrites compared to control neurons (Secondary: control: 5.5 ± 0.5; Fzd5_NP_: 8.17 ± 0.5, P<0.001; Tertiary: control: 1.13 ± 0.13; Fzd5_NP_: 5.53 ± 0.17, P<0.001) and compared to polarized neurons (secondary: Fzd5_P_: 6.55 ± 1.33; Fzd5_NP_: 8.17 ± 0.5, P<0.001; Tertiary: Fzd5_P_: 2.45 ± 0.07; Fzd5_NP_: 5.53 ± 0.17, P<0.001) (Figure 6E). Fzd5 overexpressing neurons also had significantly more branching points than control neurons (P<0.01) (Figure 6F). For further analysis of dendritic arborization, Sholl analysis was carried out. Sholl analysis allows to determine the complexity of the dendritic arbor using a semiautomated program which count the number of dendrites that intersect progressive circumferences drawn at radial distances of 5 µm from the soma [33]. GFP imaging was used for Sholl analysis (Figure S5A). For this analysis neurons that showed a polarized and non-polarized phenotype were considered. A significant increase between 10-30 µm from the soma was determined in Fzd5-overexpressing neurons (Figure S5B), but no difference was observed in the total area of the neurons (data not shown), indicating that Fzd5 had an effect on the number of neurite ramifications without affecting the size of the neurons. These results suggest that overexpression of Fzd5 and/or the somato-dendritic presence of Fzd5 induces changes in dendritic morphogenesis of neurons at stages 3 and 4.

### The inhibition of JNK partially prevented neuronal alterations induced by Fzd5 overexpression

**Figure 7:**
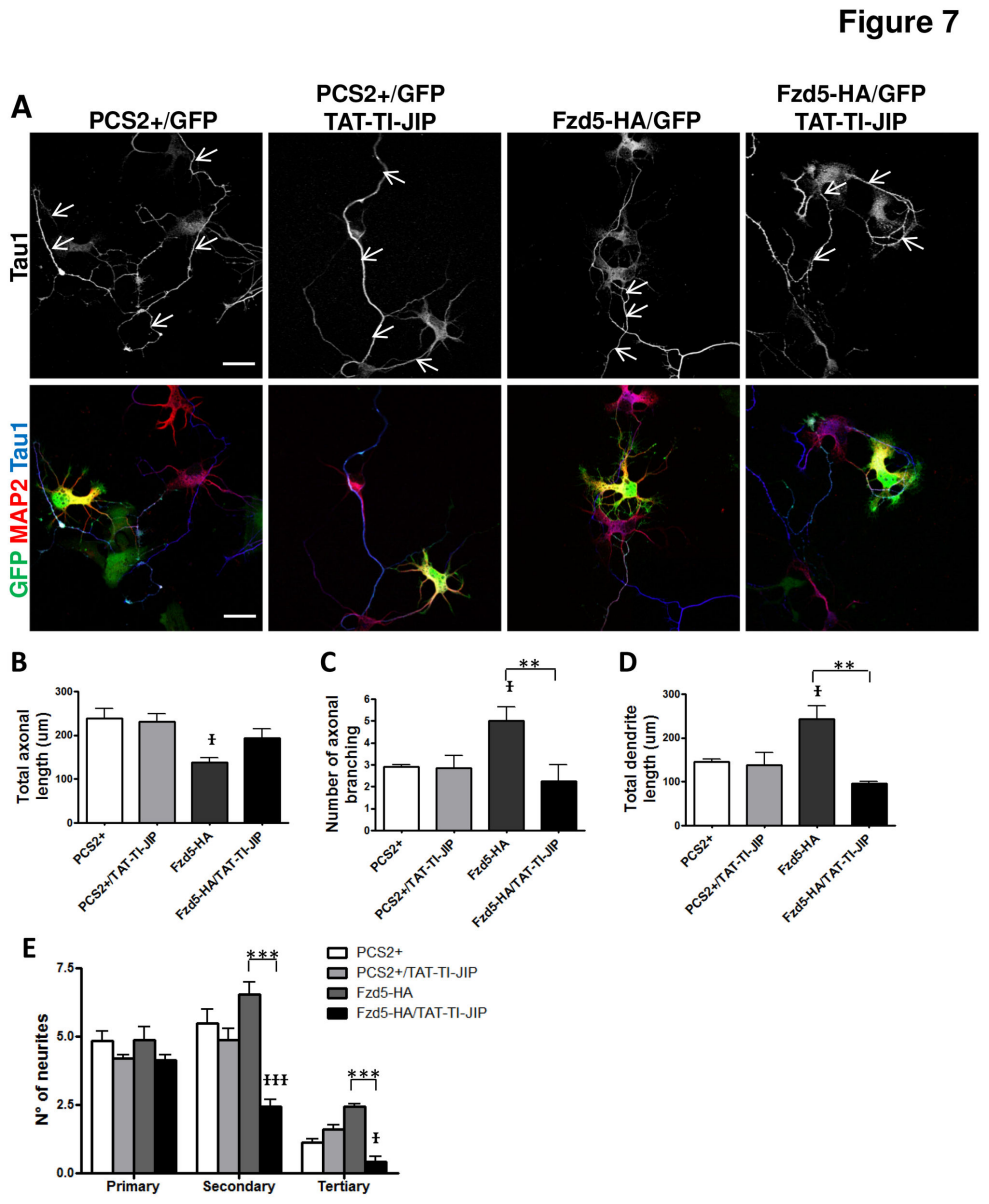
Effect of Fzd5 overexpression on neuronal morphology is partially prevented by a JNK inhibitor. **A**. Representative images showing hippocampal neurons, transfected with the empty vector PCS2+ plus GFP as a control or Fzd5-HA plus GFP, at 2 DIV, and treated with or without the JNK inhibitor, TAT-TI-JIP. Immunodetection of the axonal marker Tau1 (upper panels) and merge (lower panels) are shown. GFP was used to detect transfected neurons and white arrows mark the quantified axons. **B**, **C**. Quantification of total axonal length (B) and axonal branching (C) of control and Fzd5-overexpressing polarized neurons, with or without treatment. Neurons overexpressing Fzd5 with polarized axonal markers showed a significant increase in axonal branching and a significant decrease in axonal length compared to control and treated control neurons. These effects were prevented by TAT-TI-JIP treatments. **D**, **E**. Quantification of total dendritic length (D) and primary, secondary and tertiary dendritic branching (E) of control and Fzd5-overexpressing polarized neurons, with or without TAT-TI-JIP treatment. Neurons overexpressing Fzd5 showed a significant increase in total dendrite length and in the number of tertiary neurites compared to that of the control and treated control neurons. TAT-TI-JIP prevented these effects and in fact decreased the number of secondary and tertiary neurites. Scale bar: 20 µm. * p< 0.05; ** p< 0.01; *** p< 0.001; τ, significantly different from the control; τ p< 0.05; ττ p< 0.01; τττ p< 0.001. Error bars indicate standard error of the mean of three independent experiments for the treated neurons and five independent experiments for the neurons without treatment.

As shown in this work, the effects of Fzd5 overexpression on neuronal polarity were partially prevented by the JNK inhibitor, TAT-TI-JIP, but no by the Rho inhibitor (Figure 6). Then, it was investigated whether the effects of Fzd5 on neuronal morphology were also JNK-dependent. Neurons at 2 DIV were co-transfected with Fzd5-HA plus GFP or with the empty vector PCS2+ plus GFP as a control, and after 2 h of transfection, neurons were treated with or without 1 µM of the JNK inhibitor, TAT-TI-JIP, or 37µM of the Rho inhibitor, Y27632. Twenty-four hours later, neurons were fixed and immunostained for Tau1 and MAP2 and the axonal length and branching were analyzed (Figures 7A, S6A, axon is indicated with a white arrow). The concentrations used for both inhibitors had no effect on the variables analyzed in the present work when applied alone (Figures 5, 6 and S6). The decrease in axonal length in neurons overexpressing Fzd5 (44.5 %) was partially prevented by TAT-TI-JIP treatment (29.1 %) (Figure 7B). The increase in axonal branching from 3.21 ± 0.31 to 5.01 ± 0.56, observed in neurons transfected with Fzd5, was also prevented by TAT-TI-JIP (2.27 ± 0.73; P<0.01) and Y27632 (1.58 ± 0.46; P<0.05) (Figures 7C, S6C). Overexpression of Fzd5 also induced an increase in total dendritic length (Figure 7D), and in the number of tertiary neurites (Figure 7E) compared to control neurons. All these changes were prevented in neurons treated with the TAT-TI-JIP, which showed all parameters very similar to control neurons, and also showed a decrease in the number of secondary neurites from 5.31 ± 0.4 to 2.45 ± 0.26 (Figure 7D, E). On the other hand, treatment with Y27632 did not prevent these changes (Figure S6D, E). As almost no effects were observed with the Rho inhibitor, the capacity of Y27632 to inhibit Rho activity was corroborated using a higher concentration able to induce morphological changes *per se*. In the above described experiments 37 µM Y27632 was used as working concentration since at this concentration the inhibitor has no visible effects on the neurons, but it has been described to be capable of inhibiting the effect of Rho activation on axon formation [50]. On the other hand, treatment with 100 µM Y27632 alone leads to an increase in axonal growth [53]. So, neurons were treated with 100 µM Y27632 2 h after transfection with the empty vector and axonal length was analyzed. An increase in axonal length from 263.63 ± 32.13 to 331.8 ± 37.59 µm (data not shown) was observed in neurons treated with the inhibitor, indicating that Y27632 was indeed working. Taken together, these results suggest that changes in morphology induced by Fzd5 overexpression may involve Wnt/JNK signaling activation. 

## Discussion

It has been shown that the Wnt signaling pathway is involved in different developmental processes, including hippocampal development [54] and dendritic and axonal morphogenesis [12,17,24,55,56], but little is known about the Wnt receptors involved in these processes. We studied the Fzd5 receptor and determined that it is expressed early in cultured hippocampal neurons where it is distributed in the growth cones of immature neurites, and during neural development its localization changes localizing mainly in the axonal growth cone. Overexpression of Fzd5 induced the loss of the polarized distribution of the receptor and axonal markers, which were distributed through the entire neuron, a signal of impaired neuronal polarization. This loss of neuronal polarity was dependent on the maturation of the culture. Similarly, silencing of Fzd5 by shRNA also induced impaired neuronal polarity where no axonal marker expression was detected. In neurons showing axonal development, the overexpression of Fzd5 generated changes in axonal and dendritic morphology. All changes generated by Fzd5 overexpression were prevented partially by the JNK inhibitor, TAT-TI-JIP.

### Fzd5 is early expressed during hippocampal development

We observed that the expression of Fzd5 was higher on E18 than on postnatal hippocampus, so we decided to study Fzd5 expression in earlier development. It has been described that Fzd5 is expressed on retinal progenitors of *Xenopus*, where controls its neurogenic potential [29]. In mouse, Fzd5 is expressed in the intestinal crypts where mediates differentiation of Paneth cells [57] and is also expressed during retinal formation [27]. All the evidence that implicates Fzd5 on neuronal proliferation and differentiation are in accordance with our results. We observed that the peak of Fzd5 expression was on E17, coincident with the peak of pyramidal neurons generation in the dentate gyrus [58,59]. 

In the protocol described for the generation of hippocampal cultures [35], neurons are obtained from rats in late embryonic stages (E18 - E19) because the pyramidal neurons are completely generated, but the dentate granule neurons starts to generate later on development [58,59], allowing to obtain a homogeneous culture. Also, the neurons generated on that period are the ones that best survive on culture [58]. That is the reason why, we used rats at embryonic day E18 for the isolation and culture of hippocampal neurons.

### Fzd5 expression and distribution in growth cones suggests a role in the establishment of neuronal polarity

We determined that Fzd5 is expressed during the stages of neurite growth and axonal development, and localizes to the distal region of growth cone filopodia. At stage 2, neurons presented Fzd5 in the growth cone of most neuronal processes, but as development progressed, Fzd5 started to concentrate its expression in the peripheral zone of the axonal growth cone and decreased in dendrites. It has been described that in the first developmental stages, all neurites are similar in length and proteins are distributed symmetrically to each of them. Then, before the development of the axon, the proteins become concentrated to the neurite with a more dynamic actin cytoskeleton and more stable microtubules, neurite that will become the axon [37,60,61]. Proteins involved in neuronal polarity, such as the polarity Par3/Par6/aPKC complex, the monomeric GTPase Cdc-42 [62] and IGF-1R, are enriched in the axonal growth cone [63], as was determined for Fzd5. Therefore, this evidence reinforces the idea that enrichment of Fzd5 at the tip of the developing axon, may be an indicative of Fzd5 participating in neuronal polarity. 

Sahores, et.al. (2010) described a different distribution for Fzd5, characterized by the presence of Fzd5 mainly in dendrites. However, their study was focused on the role of Fzd5 in synaptogenesis and did not analyze earlier stages of neuronal development. There is evidence of proteins that change their distribution pattern after the establishment of the axon, like Ror1 and Ror2 receptors. During stage 2 and 3 of neuronal development, Ror1 and Ror2 receptors are concentrated in the growth cones and during dendrite development, the localization of both receptors is changed and the receptors immunoreactivity is detected mainly in the somato-dendritic compartment through all the dendrites [64]. Like Ror receptors, Fzd5 could be concentrated in different neuronal compartments during distinct stages of development, which may correlate with the different functions of the receptor. 

During axonal outgrowth, receptors present at the growth cone can be localized to filopodia, leading to rapid changes in actin and microtubule dynamics, which ultimately promotes axonal elongation [4,65]. The distribution of Fzd5 at distal filopodia in growth cones suggests that Fzd5 might be acting concertedly with Wnt-5a and/or Wnt-7a, since both have been described as Fzd5 ligands [26,27,28]. Wnt-5a increases axonal growth, while Wnt-7a induces shorter axons and both ligands stimulate an increase in axonal branching [12,14,15]. This is supported by the fact that Fzd5 overexpression induced changes in axonal and dendritic morphology in neurons that have already developed the axon. It is worth noting that Fzd5 decreases its expression in dendrites during development and that overexpression of Fzd5 leads to the expression of Fzd5 in the whole cell, so we cannot discard the possibility that the effects seen in dendritic development are generated because of a change in Fzd5 distribution.

### Fzd5 might have a role in polarity establishment

On one hand, we observed that Fzd5 over-expression at 2 DIV induced a loss of neuronal polarity in 50% of neurons. Specifically we observed that overexpression of Fzd5 resulted in a loss of the polarized distribution of Tau1 and MAP1B-P, these axonal markers were now distributed through the entire neuron. On the other hand, the diminished expression of Fzd5 obtained with shRNA induced a loss of axonal markers. These results suggest the existence of a threshold for Fzd5 expression, which is necessary for the expression and distribution of axonal markers during the establishment of neuronal polarity. Similarly, other receptors also contribute to the development of the axon. IGF-1 receptor inhibition impairs the correct polarization of neurons [63].

Gain-of-function experiments suggest that the Fzd5 effects would be more important before the onset of axonal acquisition. We showed that Fzd5 overexpression alters neuronal polarization before the establishment of polarity, but once the axon starts to develop, Fzd5 is no longer able to impair polarity, possibly because of the formation of the axonal initial segment which acts as a diffusion barrier that prevents axonal protein transport away from the axon [66].

The analysis carried out in the present study did not consider the neuronal subpopulations, but at the first developmental stages Fzd5 was expressed almost in 100 % of the neurons. It would be very interesting to determine in future experiments if the effects observed by Fzd5 over-expression is specific to one population of hippocampal neurons.

### The effects of Fzd5 are partially prevented by the inhibition of JNK

The activation of GSK-3β could lead to modifications on the actin cytoskeleton thought target proteins like CRMP2. CRMP2 is a protein that associates with tubulin heterodimers promoting axonal development, and once that is phosphorylated by GSK-3β induces growth cone collapse [67,68]. Our results showed that Fzd5 failed on activating GSK-3β and CRMP2. 

Finally, we determined that the impaired polarity induced by Fzd5 overexpression and the changes in morphology induced by augmented expression of Fzd5 in neurons, that have already developed an axon, were prevented partially by treatment with the JNK inhibitor, TAT-TI-JIP. And that Fzd5 overexpressed in HEK293 cells activated JNK, which was demonstrated by the increase in p-JNK and p-c-Jun levels. Although the increase on p-c-Jun was not significant, a tendency was observed. These results suggest that Fzd5 could be modulating the JNK cascade to generate part of the observed changes. During the extension of neuronal processes, JNK is activated distally in the axon [45] and in the tips of dendrites [47] to promote the extension of axons in stage 3 neurons and the dendrite development in stage 4 neurons, respectively. Interestingly, the same distribution was observed in this study for endogenous Fzd5 in stage 2 neurons and later on in development, only in the axon, thus Fzd5 may mediate JNK activation to promote axon extension. In Fzd5 overexpression experiments, when Fzd5 was distributed in the whole cell, the receptor could have been activating JNK in both the axon and dendrites to promote the extension of the processes in a similar extent. One of the functions of JNK is the regulation of the activity of the MAP family, such as Tau1 and MAP1BP, which are essential for axonal formation and elongation [69,70]. MAP1BP is able to interact with microtubules, stabilizing them and activating the small GTPases Rac1 and Cdc-42, which regulates actin cytoskeleton dynamics [46]. Our results suggest that Fzd5 could be activating the Wnt/JNK cascade, inducing the activation of Rac and the concomitant activation of JNK in the axon, which is translated to changes in cytoskeleton dynamics and to morphological changes. Is important to highlight that the inhibition of JNK prevented only partially the effects generated by Fzd5 overexpression so, Fzd5 might be activating other signaling cascades that could be responsible of the rest of the effects. Further studies will help to elucidate the precise mechanisms involved in Fzd5 modulation.

## Supporting Information

Figure S1 **Fzd5 is expressed in the hippocampus, at least, during latest embryonic development.** Detection of Fzd5 protein levels in hippocampi E17 - 19 homogenates. The greater receptor levels were observed during E17 and diminished concomitantly with the progression of embryonic development.
(TIF)

Figure S2 **Specificity of Fzd5 immunodetection.** Immunodetection of Fzd5 at 2 DIV hippocampal neurons with different antibodies. With the antibodies against the C-terminal and N-terminal regions of the receptor, a positive staining for Fzd5 was observed at the tips of the filopodia, co-distributed with phalloidin, indicating that there is a specific labeling for the receptor. In the lower panels a magnification of the filopodia is shown. Scale bar: 20 µm.
(TIF)

Figure S3 **Augmented Fzd5 expression by Fzd5 transfection.** Immunodetection of Fzd5 and the neuronal marker β-III-tubulin in 3 DIV neurons co-transfected with Fzd5-HA plus GFP. Fzd5 staining is augmented and distributed through the whole cell in Fzd5-overexpressing neurons (yellow arrow) compared with control neurons (white arrow). Scale bar: 20 µm.
(TIF)

Figure S4 **Diminished Fzd5 expression by shRNA.** **A**. Immunodetection of Fzd5 and the neuronal marker β-III-tubulin in 2 DIV neurons transfected with shRNA for Fzd5. Fzd5 staining is lost in shRNA Fzd5 neurons, GFP positives (yellow arrow), compared with control neurons (white arrow). **B**. Detection of Fzd5 protein levels in total homogenates of control or shRNA-transfected PC12 cells. The receptor levels are diminished in shRNA-transfected cells. GFP was used as a transfection control and actin was used as loading control. Asterisk indicates a non-specific band.
(TIF)

Figure S5 **Sholl analysis of Fzd5 overexpressing neurons.** **A**. Representative images showing hippocampal neurons 24 h after transfection with the empty vector PCS2+ plus GFP as a control or Fzd5-HA plus GFP at 2 DIV. Scale bar: 20 µm. **B**. Sholl analysis of hippocampal neurons. Neurons overexpressing Fzd5 showed a significantly increased number of intersections near the soma. * p< 0.05; ** p< 0.01. Error bars indicate standard error of the mean of three independent experiments.
(TIF)

Figure S6 **The Rho inhibitor, Y27632, failed to prevent neuronal alterations induced by Fzd5 overexpression.** **A**. Representative images showing hippocampal neurons transfected with the empty vector PCS2+ plus GFP as control or Fzd5-HA plus GFP, treated with or without the Rho inhibitor, Y27632. Immunodetection of the axonal marker Tau1 (upper panels) and merge (lower panels) are shown. GFP was used to detect transfected neurons. White arrows mark the quantified axons. **B**, **C**. Quantification of total axonal length (B) and axonal branching (C) of control and Fzd5-overexpressing polarized neurons, with or without Y27632 treatment. Neurons overexpressing Fzd5 with polarized axonal markers showed a significant increase in axonal branching and a significant decrease in axonal length compared to the control and treated Fzd5-overexpressing neurons. Only the effect of Fzd5 on the number of axonal branching was prevented by Y27632. **D**, **E**. Quantification of total dendritic length (D) and primary, secondary and tertiary dendritic branching (E) of control and Fzd5-overexpressing polarized neurons with or without treatment. Neurons overexpressing Fzd5 showed a significant increase in total dendrite length and in the number of tertiary neurites compared to control and treated control neurons. Y27362 treated, Fzd5-overexpressing neurons also showed an increase in the total dendritic length, but no change in the number of tertiary neurites was observed. Scale bar: 20 µm. * p< 0.05; ** p< 0.01; τ, significantly different from the control; τ p< 0.05. Error bars indicate standard error of the mean of three independent experiments for the treated neurons and five independent experiments for the neurons without treatment.
(TIF)
