# Supplementary figures and images for: Frizzled-5 Receptor Is Involved in Neuronal Polarity and Morphogenesis of Hippocampal Neurons
Source: PLoS One. 2013 Oct 18;8(10):e78892. doi: 10.1371/journal.pone.0078892 (PMC3800132; doi:10.1371/journal.pone.0078892)

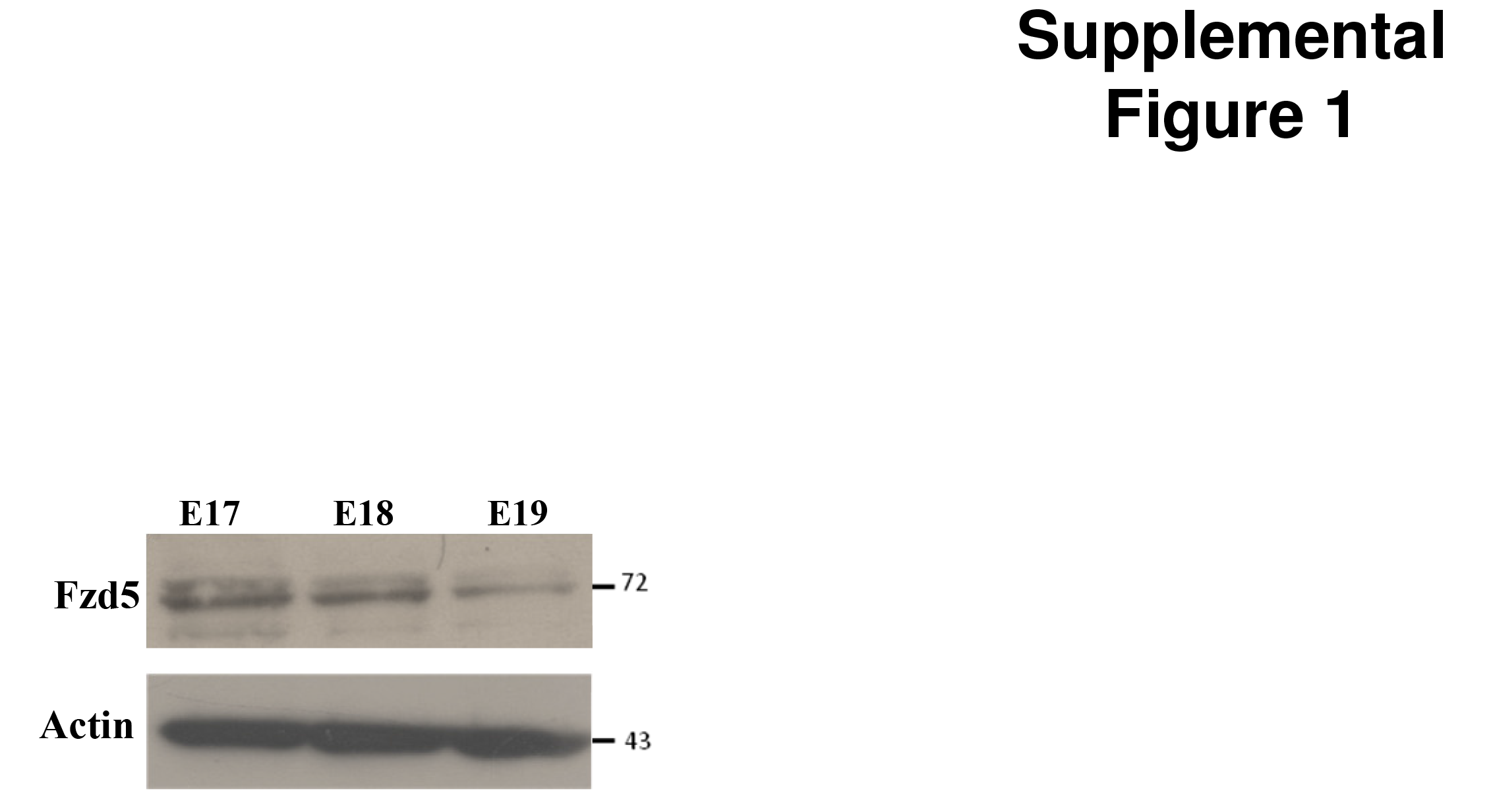

Supplement: Figure S1 — Fzd5 is expressed in the hippocampus, at least, during latest embryonic development. Detection of Fzd5 protein levels in hippocampi E17 - 19 homogenates. The greater receptor levels were observed during E17 and diminished concomitantly with the progression of embryonic development. (TIF) [file pone.0078892.s001.tif]

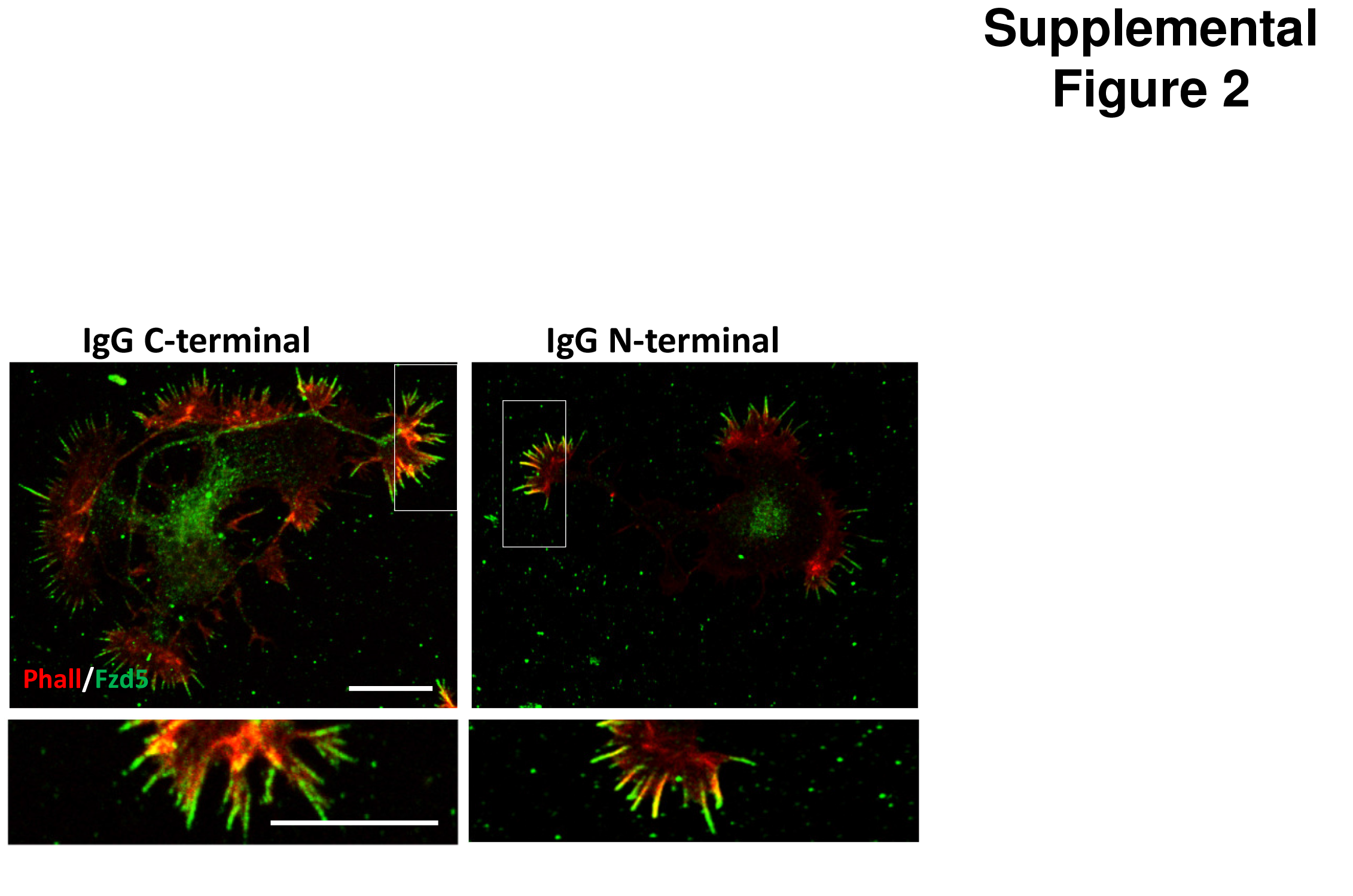

Supplement: Figure S2 — Specificity of Fzd5 immunodetection. Immunodetection of Fzd5 at 2 DIV hippocampal neurons with different antibodies. With the antibodies against the C-terminal and N-terminal regions of the receptor, a positive staining for Fzd5 was observed at the tips of the filopodia, co-distributed with phalloidin, indicating that there is a specific labeling for the receptor. In the lower panels a magnification of the filopodia is shown. Scale bar: 20 µm. (TIF) [file pone.0078892.s002.tif]

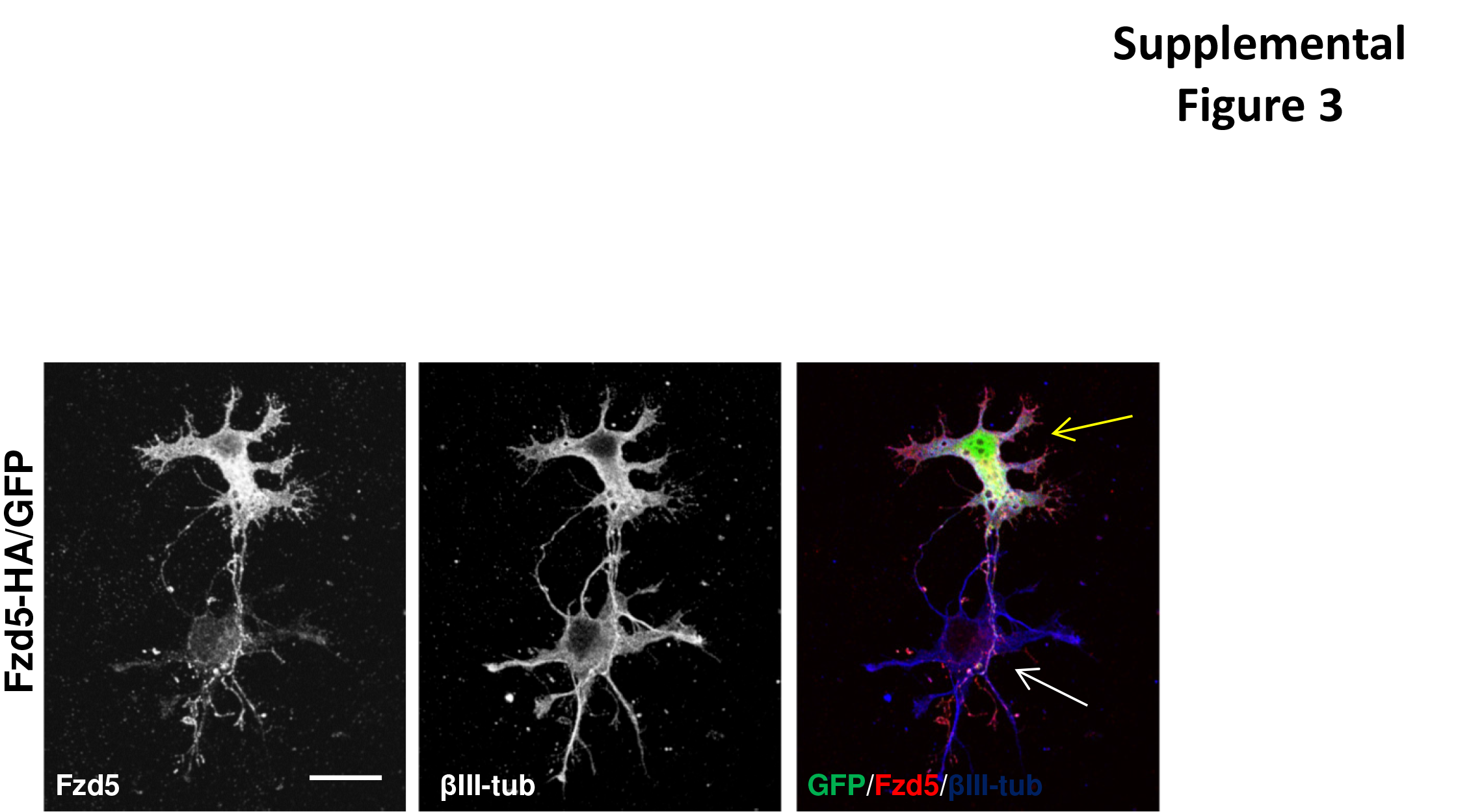

Supplement: Figure S3 — Augmented Fzd5 expression by Fzd5 transfection. Immunodetection of Fzd5 and the neuronal marker β-III-tubulin in 3 DIV neurons co-transfected with Fzd5-HA plus GFP. Fzd5 staining is augmented and distributed through the whole cell in Fzd5-overexpressing neurons (yellow arrow) compared with control neurons (white arrow). Scale bar: 20 µm. (TIF) [file pone.0078892.s003.tif]

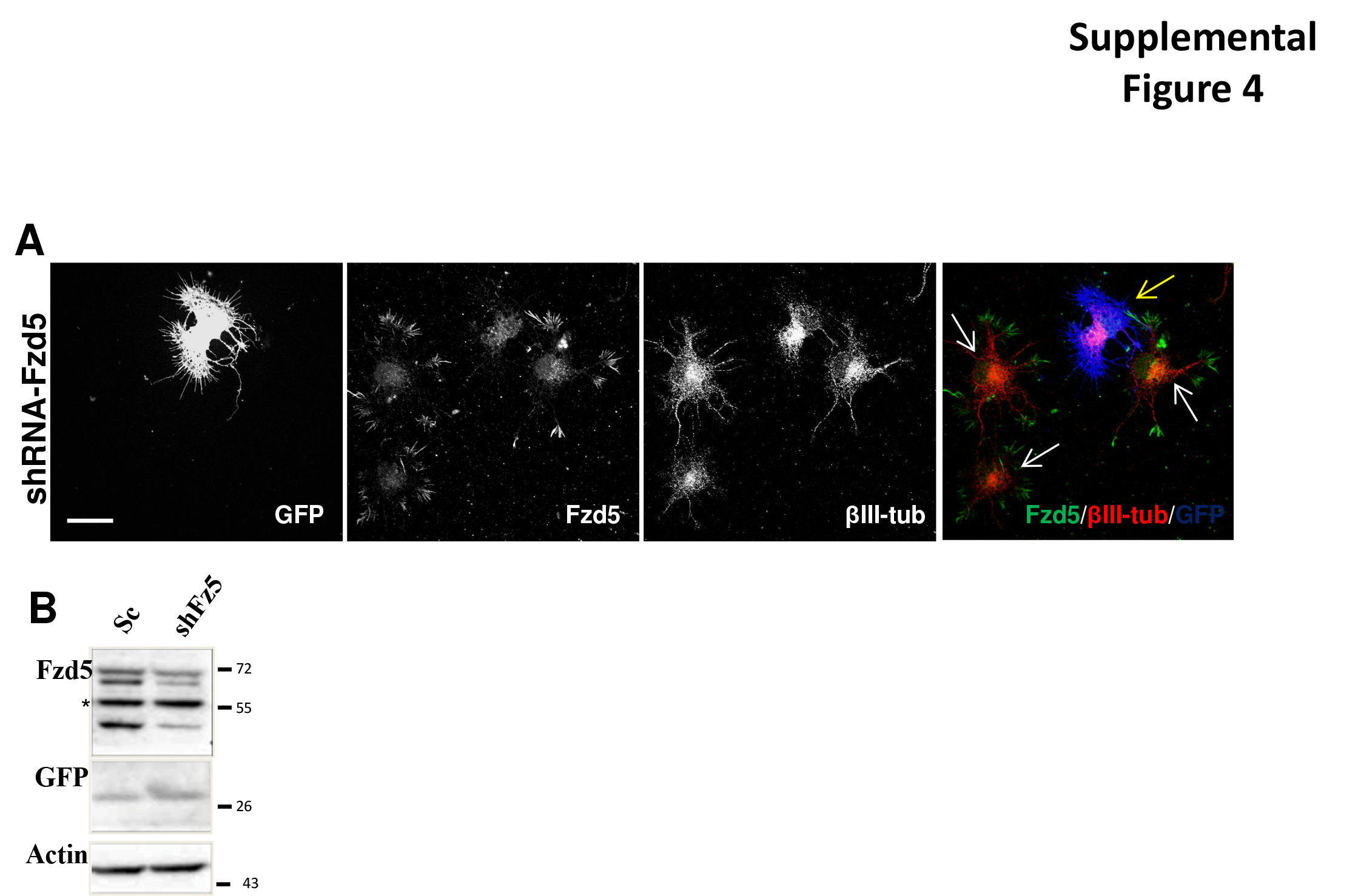

Supplement: Figure S4 — Diminished Fzd5 expression by shRNA. A. Immunodetection of Fzd5 and the neuronal marker β-III-tubulin in 2 DIV neurons transfected with shRNA for Fzd5. Fzd5 staining is lost in shRNA Fzd5 neurons, GFP positives (yellow arrow), compared with control neurons (white arrow). B. Detection of Fzd5 protein levels in total homogenates of control or shRNA-transfected PC12 cells. The receptor levels are diminished in shRNA-transfected cells. GFP was used as a transfection control and actin was used as loading control. Asterisk indicates a non-specific band. (TIF) [file pone.0078892.s004.tif]

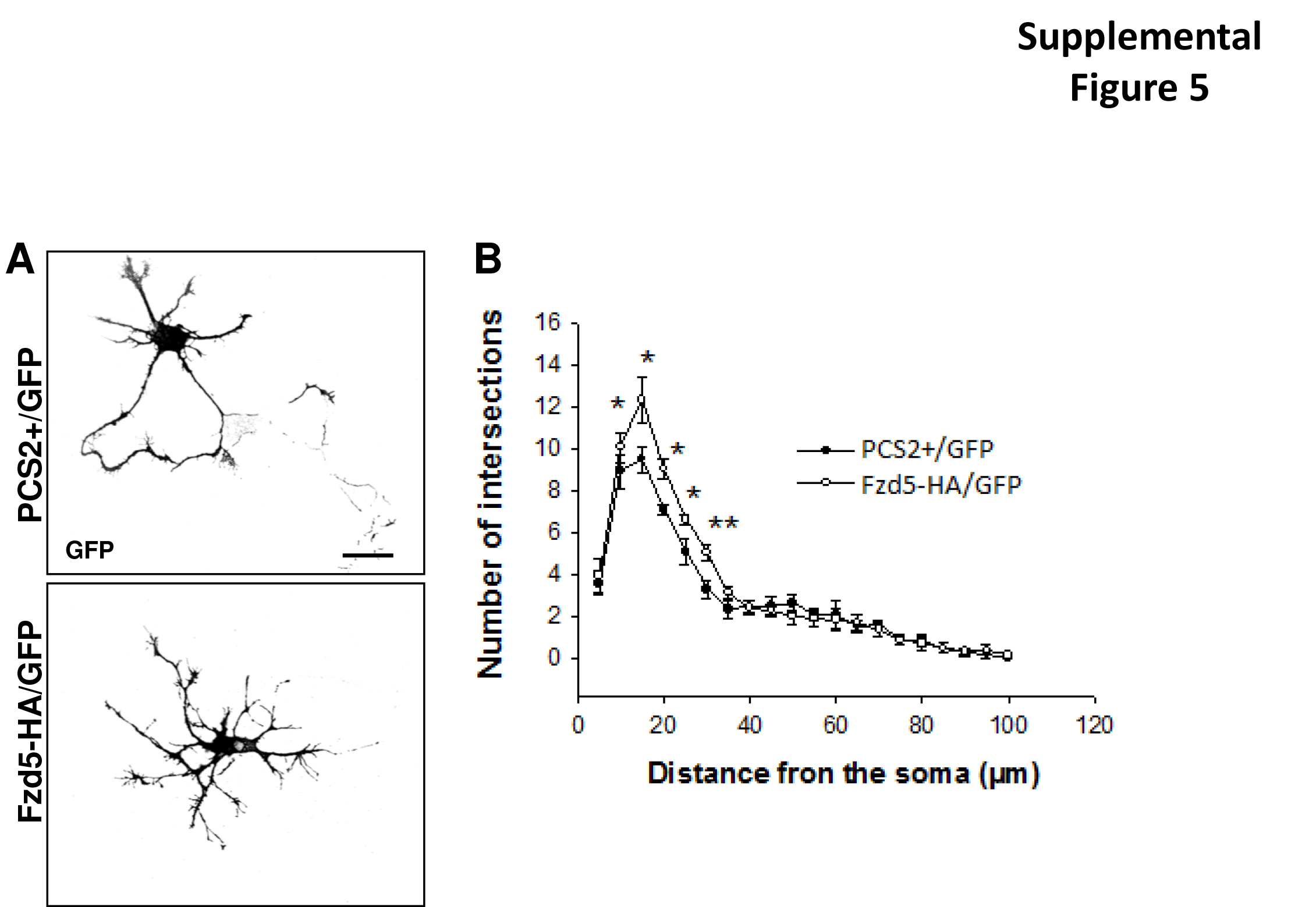

Supplement: Figure S5 — Sholl analysis of Fzd5 overexpressing neurons. A. Representative images showing hippocampal neurons 24 h after transfection with the empty vector PCS2+ plus GFP as a control or Fzd5-HA plus GFP at 2 DIV. Scale bar: 20 µm. B. Sholl analysis of hippocampal neurons. Neurons overexpressing Fzd5 showed a significantly increased number of intersections near the soma. * p< 0.05; ** p< 0.01. Error bars indicate standard error of the mean of three independent experiments. (TIF) [file pone.0078892.s005.tif]

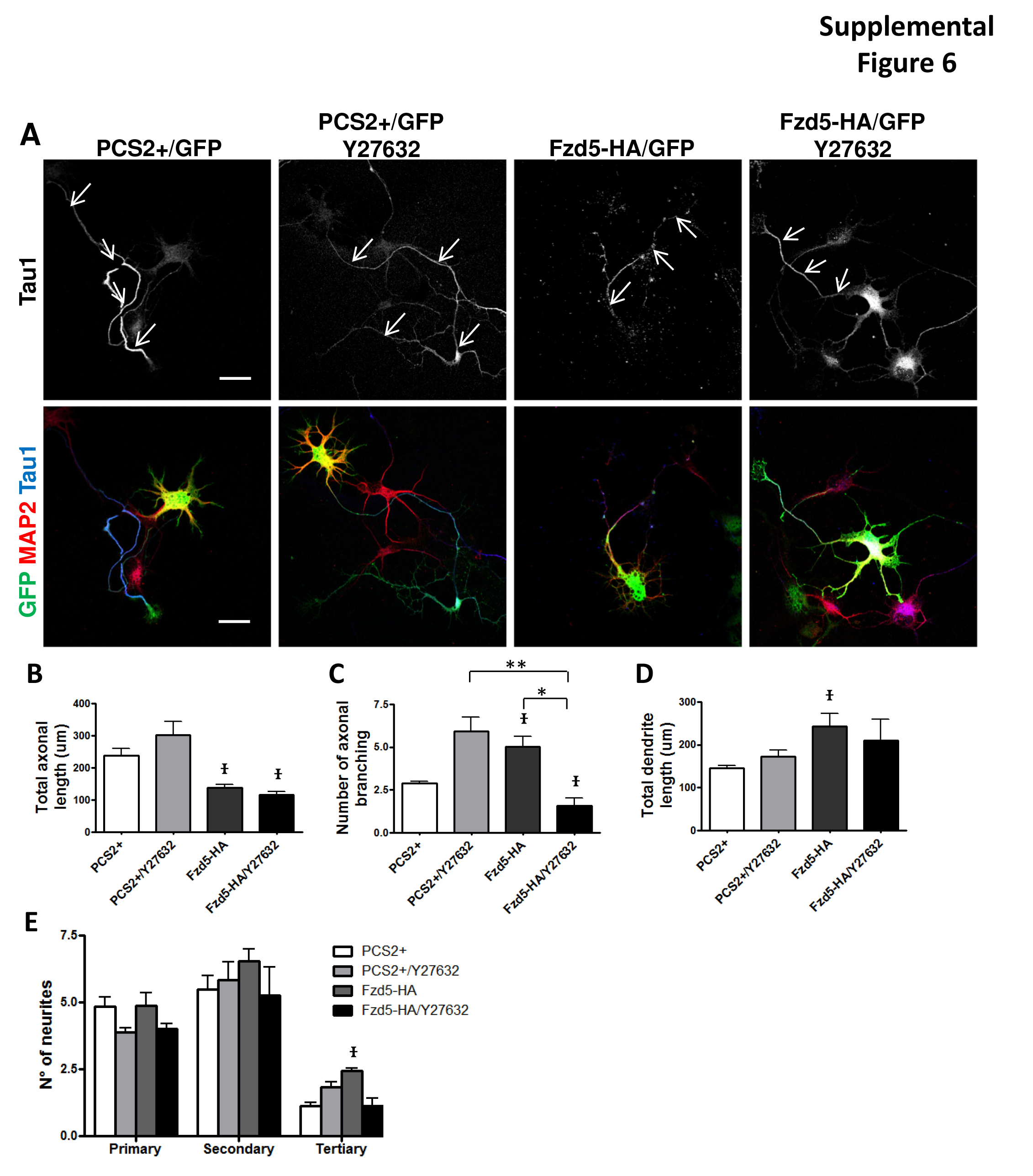

Supplement: Figure S6 — The Rho inhibitor, Y27632, failed to prevent neuronal alterations induced by Fzd5 overexpression. A. Representative images showing hippocampal neurons transfected with the empty vector PCS2+ plus GFP as control or Fzd5-HA plus GFP, treated with or without the Rho inhibitor, Y27632. Immunodetection of the axonal marker Tau1 (upper panels) and merge (lower panels) are shown. GFP was used to detect transfected neurons. White arrows mark the quantified axons. B, C. Quantification of total axonal length (B) and axonal branching (C) of control and Fzd5-overexpressing polarized neurons, with or without Y27632 treatment. Neurons overexpressing Fzd5 with polarized axonal markers showed a significant increase in axonal branching and a significant decrease in axonal length compared to the control and treated Fzd5-overexpressing neurons. Only the effect of Fzd5 on the number of axonal branching was prevented by Y27632. D, E. Quantification of total dendritic length (D) and primary, secondary and tertiary dendritic branching (E) of control and Fzd5-overexpressing polarized neurons with or without treatment. Neurons overexpressing Fzd5 showed a significant increase in total dendrite length and in the number of tertiary neurites compared to control and treated control neurons. Y27362 treated, Fzd5-overexpressing neurons also showed an increase in the total dendritic length, but no change in the number of tertiary neurites was observed. Scale bar: 20 µm. * p< 0.05; ** p< 0.01; τ, significantly different from the control; τ p< 0.05. Error bars indicate standard error of the mean of three independent experiments for the treated neurons and five independent experiments for the neurons without treatment. (TIF) [file pone.0078892.s006.tif]
